# Intraoperative extracorporeal autogenous irradiated tendon grafts for functional limb salvage surgery of soft tissue sarcomas of the wrist and hand

**DOI:** 10.1186/s12957-015-0588-4

**Published:** 2015-05-12

**Authors:** Shinsuke Omori, Kenichiro Hamada, Hidetatsu Outani, Kazuya Oshima, Susumu Joyama, Yasuhiko Tomita, Norifumi Naka, Nobuhito Araki, Hideki Yoshikawa

**Affiliations:** Department of Orthopaedic Surgery, Osaka University Graduate School of Medicine, 2-2, Yamada-oka, Suita, Osaka 565-0871 Japan; Department of Orthopaedic Surgery, Osaka Medical Center for Cancer and Cardiovascular Diseases, 1-3-3 Nakamichi, Higashinari, Osaka 537-8511 Japan; Department of Pathology, Osaka Medical Center for Cancer and Cardiovascular Diseases, 1-3-3 Nakamichi, Higashinari, Osaka 537-8511 Japan

**Keywords:** Limb salvage operation, Hand, Wrist, Intraoperative extracorporeal autogenous irradiated tendon and bone graft

## Abstract

**Background:**

In patients with soft tissue sarcoma of the wrist and hand, limb salvage operation is extremely challenging for surgeons in attempting a complete tumor resection with negative surgical margins. In this study, we report four patients with soft tissue sarcoma of the wrist and hand treated by limb salvage operation with intraoperative extracorporeal autogenous irradiated tendon grafts.

**Methods:**

The patients were all male, and the mean age at the time of surgery was 45 years. Histological diagnoses included clear cell sarcoma in two patients, synovial sarcoma in one, and angiosarcoma in one. All four patients had high grade tumors, wherein three had American Joint Committee on Cancer (AJCC) stage III disease and one with AJCC stage IV disease. The tumors were resected en bloc with involved tendons. The tendons were isolated from the resected tissues, irradiated *ex vivo*, and re-implanted into the host tendons. In one patient, the bone was resected additionally because of tumor invasion to the bone. Hand function was evaluated using Musculoskeletal Tumor Society (MSTS) rating system.

**Results:**

Of the four patients, three died of distant metastatic disease. The remaining patient lives and remains disease-free. The mean follow-up period was 33 months. One patient had local recurrence outside the irradiated graft at 20 months after surgery. The functional rating was 22. Lower scores were seen in patients with reconstruction of flexor tendons than extensor tendons.

**Conclusions:**

Limb salvage operation with intraoperative extracorporeal autogenous irradiated tendon grafts is an acceptable method in selected patients with soft tissue sarcoma of the wrist and hand.

## Background

Soft tissue sarcomas are uncommon and account for less than 1% of all malignant tumors [1,2]. They occur most commonly on the trunk and lower extremities and only 10% to 15% on the upper extremities [3]. Sarcomas of the forearm and hand account for less than 3% of upper limb tumors [4]; therefore, they are very rare [5,6].

The highest priority in surgical management of all soft tissue sarcomas is to achieve complete excision with negative margins. However, in the wrist and hand, it is difficult to achieve wide surgical margins and to preserve function because of the complex anatomy and limited tissue volume [6,7].

Extracorporeal irradiation of tissue followed by re-implantation has been performed to replace bone and tendon defects following removal of tumors [8-10]. This technique enables biological reconstruction with a precise fit and helps to restore the function of joints [8-10].

In four patients who had soft tissue sarcomas that involved the wrist and hand, extracorporeal autogenous irradiated tendon grafts were used in order to improve function following tumor excision. In this study, we report the results and advantages of this surgical management.

## Methods

Between the years 1994 and 2009, we treated four patients with soft tissue sarcomas of the wrist and hand by limb salvage surgery with intraoperative extracorporeal autogenous irradiated tendon graft. All patients were male with a mean age of 45 years (range, 36 to 62 years). The mean follow-up period was 33 months (range, 25 to 47 months).

All patients were treated for their primary tumors at our institutions. Preoperative staging was conducted with the use of computed tomography (CT) scans of the chest and magnetic resonance imaging (MRI) of the wrist and hand in order to define the extent of the tumor and involvement of the bone, soft tissues, particularly the neurovascular bundle, and tendon. The American Joint Committee on Cancer (AJCC) staging system was used to stage the tumors [11].

**Figure 1:**
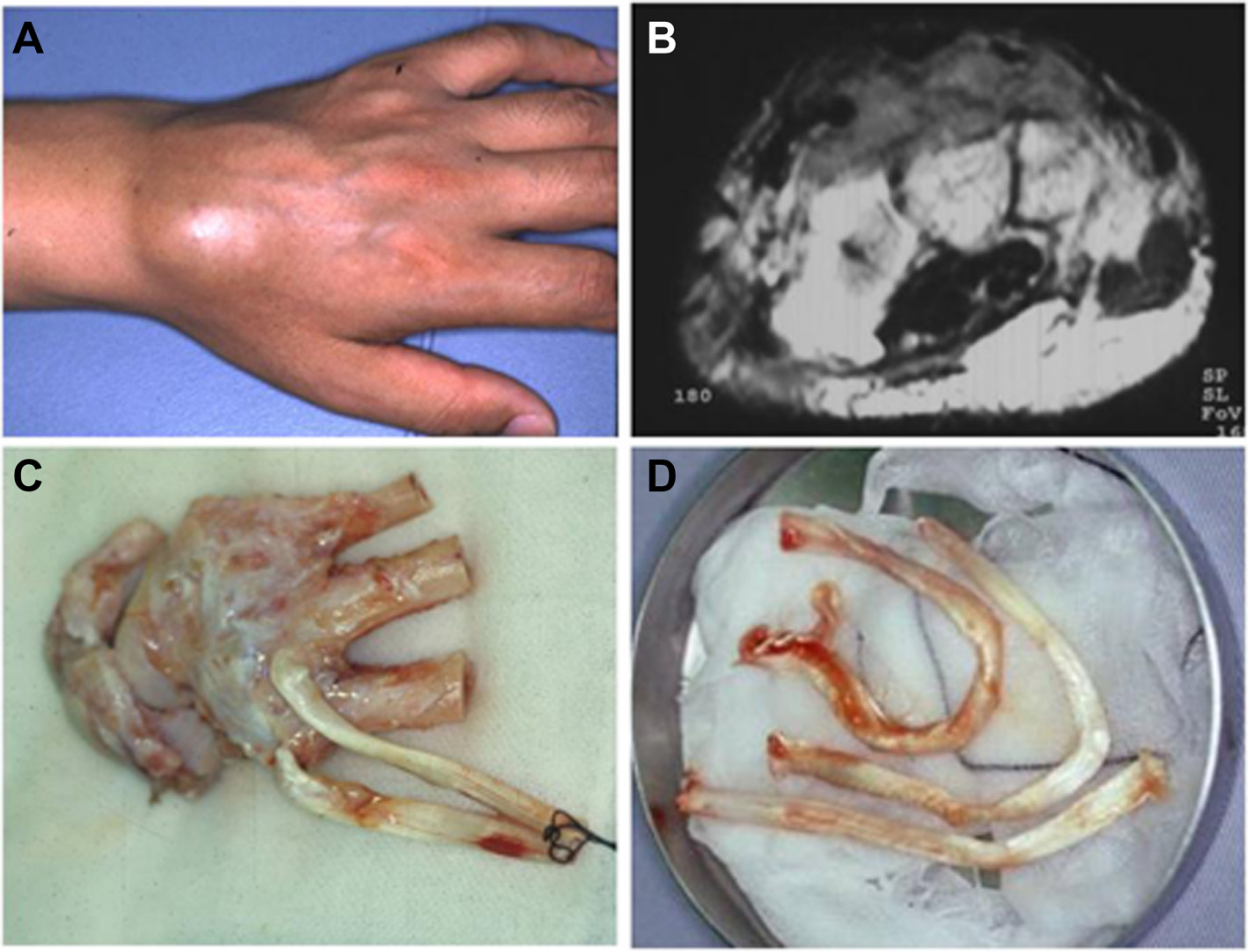
A 43-year-old male patient with clear cell sarcoma on dorsum of wrist. Clear cell sarcoma on dorsum of wrist. **(A)** A 43-year-old man with a 2-year history of a slow-growing mass on the dorsum of the left hand was shown. **(B)** MRI of the tumor was highly inhomogeneous on T2-weighted images, which was located beneath the extensor tendons and was attached to the carpal and metacarpal bones. **(C**, **D)** The tumor was resected including the carpal and metacarpal bones and extensor tendons. The resected tissues were trimmed and irradiated with 50 Gy one fraction.

**Figure 2:**
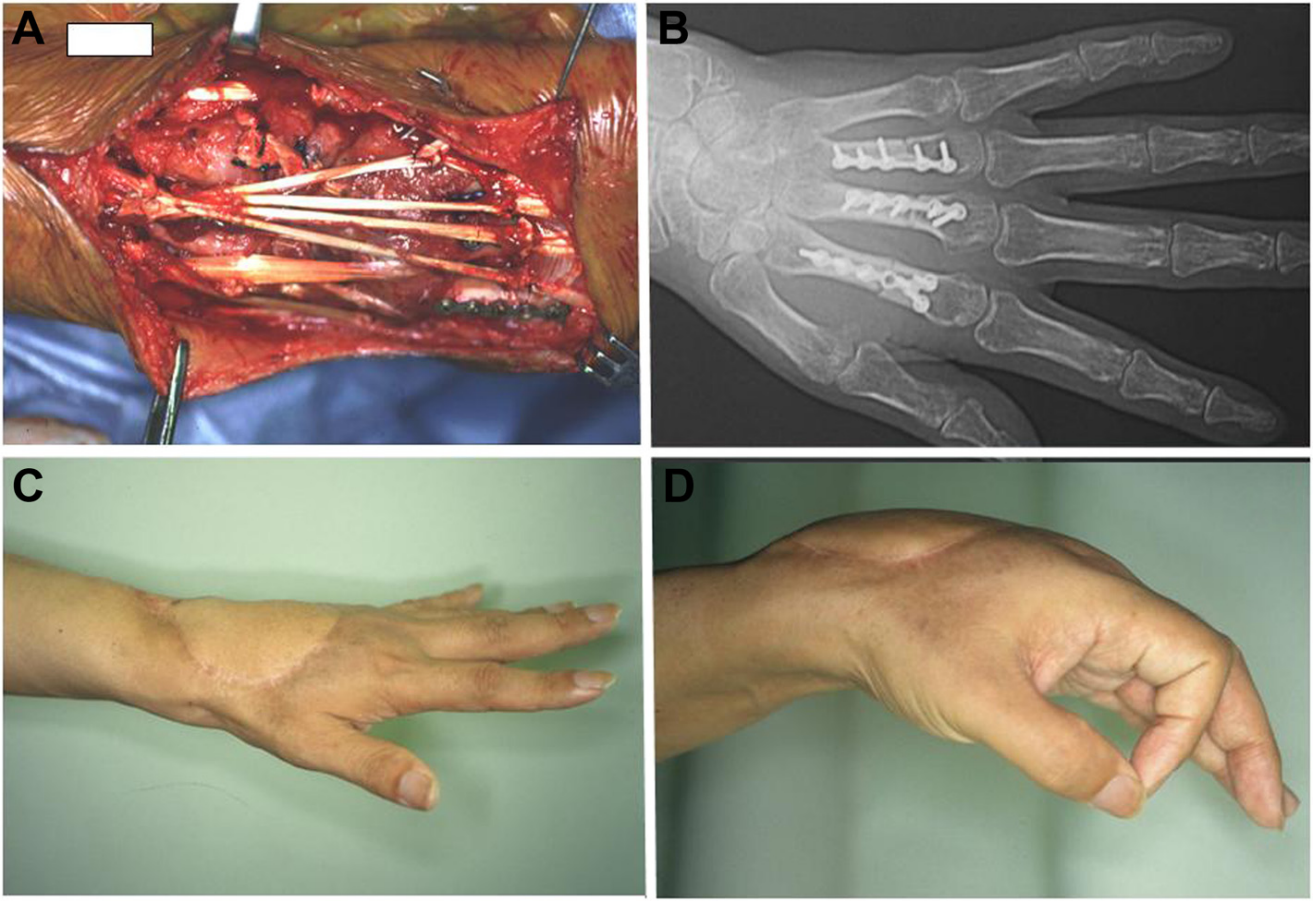
Intraoperative view and postoperative hand function. **(A)** The extensor digitorum muscles and bones were re-implanted to the original corresponding structures. **(B)** Postoperative radiograph. The metacarpal bones were fixed with the phalangeal plates. **(C**, **D)** The patient could extend his fingers and wrist due to the re-sutured tendons. MSTS rating system was 24.

The treatment procedures were as follows (Figures 1 and 2): (1) en bloc resection of the tumor with involved tendons, (2) isolation of the tumor from the resected tendons, (3) extracorporeal irradiation with 50 Gy as a single bolus dose to the isolated tendons in a plastic container, and (4) re-implantation of the irradiated tendons. Tendons were sutured to the original corresponding structures. In one patient, the metacarpal and carpal bones were resected additionally because the tumor invaded the bone (Figure 1). The irradiated bones were also re-implanted into the host bones with fixation devices.

Hand function was assessed for all patients using the Musculoskeletal Tumor Society (MSTS) functional evaluation system [12]. The MSTS score is used to assess the outcomes after tumor resection and reconstruction. In the upper extremity, the factors were composed of pain, function, emotional acceptance, hand positioning, dexterity, and lifting ability. Each factor is assigned a value of 0 to 5 with the total score ranging from 0 to 30, wherein a higher score indicates better functional outcome.

Postoperative complications during follow-up were analyzed, and local recurrence and overall survival were also evaluated.

An informed consent was obtained from all the patients who were treated with this surgery. This treatment was conducted in accordance with the Helsinki Declaration.

## Results and discussion

Clinical characteristics of the patients are summarized in Table 1. Three patients died because of complications related to pulmonary metastasis. One patient remains alive and disease-free. There were no tumor recurrences from the irradiated grafts, but recurrence occurred outside the irradiated graft at 20 months after surgery in one patient (Case 3). This local recurrence was resected, but the patient died of pulmonary metastasis at 16 months after local recurrence.

**Table 1:** **Clinical characteristics of the patients**

**Case**	**Age/sex**	**Location**	**Size (cm)/depth**	**Histology**	**Stage AJCC**	**CTx**	**RTx**	**Follow-up period**
1	43/M	Dorsal wrist	5/deep	Clear cell sarcoma	III	+	−	47
2	62/M	Dorsal wrist	7/deep	Synovial sarcoma	III	+	+	36
3	36/M	Palm	8/deep	Clear cell sarcoma	IV	+	−	25
4	38/M	Volar wrist	8/deep	Angio sarcoma	III	+	−	25

CTx, chemotherapy; RTx, radiation therapy.

**Figure 3:**
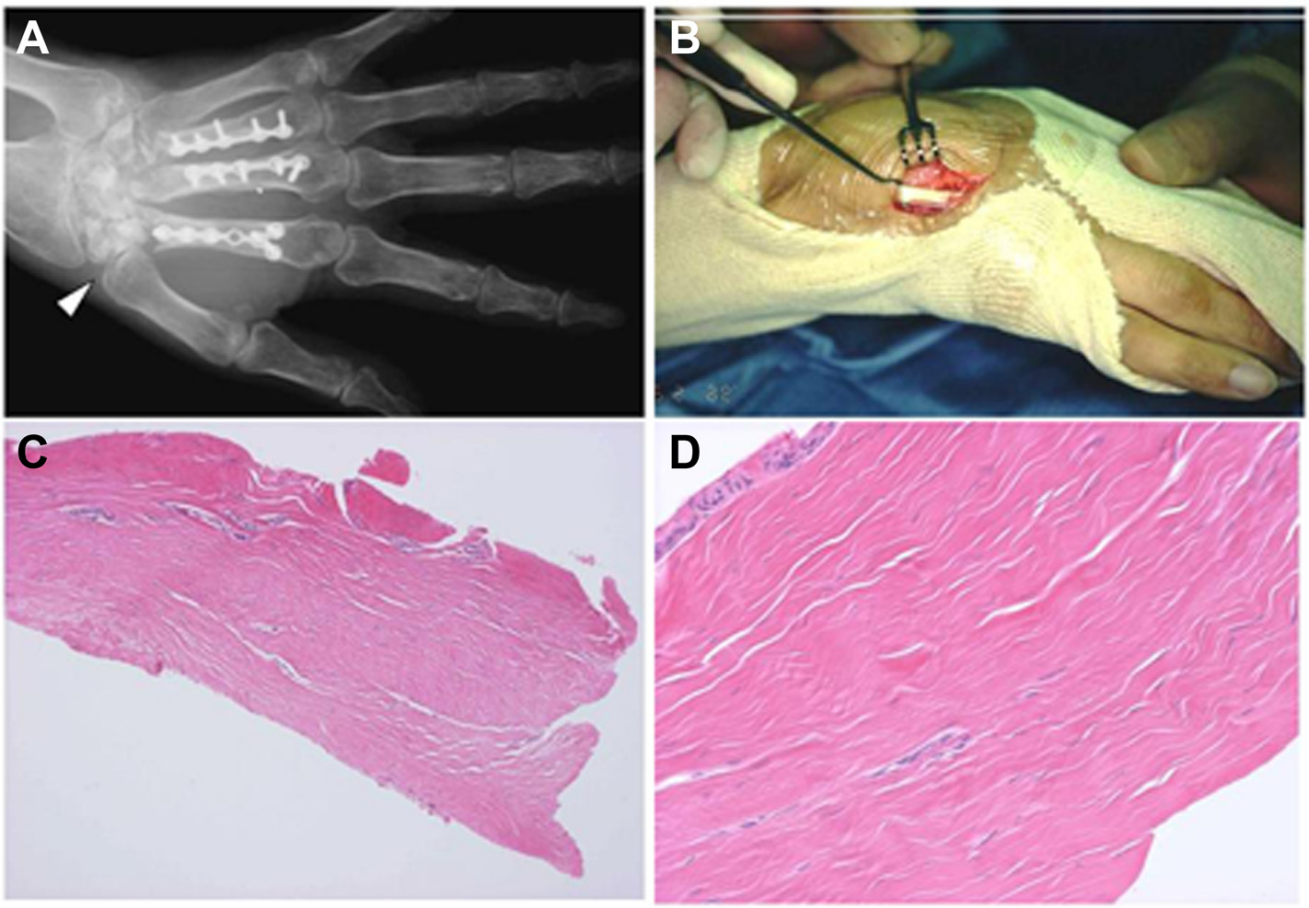
Postoperative complication and histology of irradiated tendon. **(A)** Plain radiograph. Collapse of carpal bone occurred (arrow head). **(B)** Histological examination of irradiated tendon is shown. **(C**, **D)** Histology of irradiated tendon. Fibroblast-like cells were seen within the strands of collagen that appeared to be morphologically normal.

**Figure 4:**
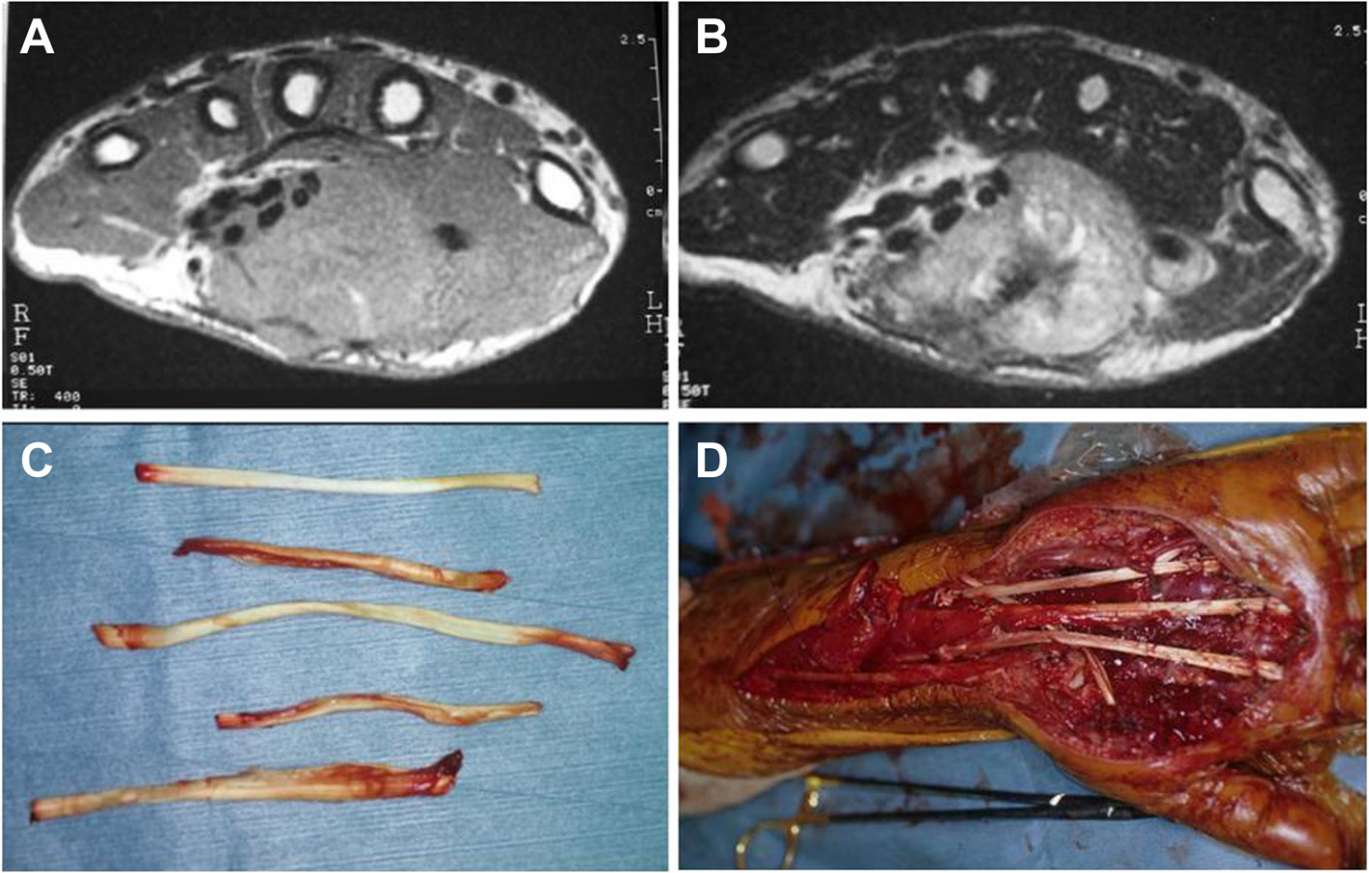
A 36-year-old male patient with clear cell sarcoma of the palm. MRI showed a mass that spanned the palmar aspect of the hand with iso-intensity on T1- **(A)** and was highly inhomogeneous on T2-weighted images **(B)** and involved the volar compartment (deep, superficial digital flexor tendon, and median nerve). The flexor tendons were stripped from the resected tumor tissues **(C)**. The irradiated flexor digitorum profundus muscle was sutured to the opponens pollicis muscle, and skin flap was used for wound closure **(D)**.

**Figure 5:**
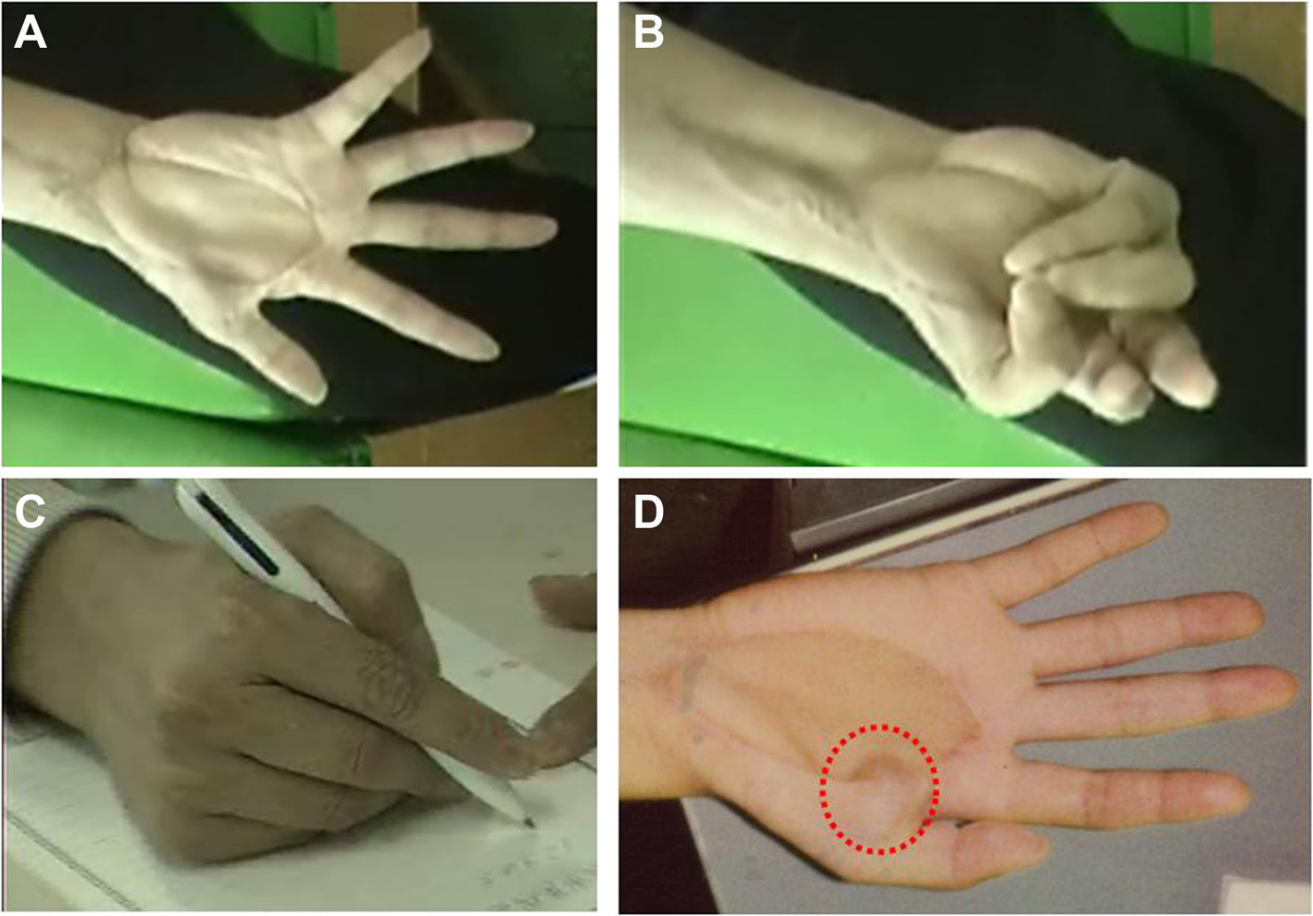
Postoperative hand function. The patient could spread out the palm **(A)** but had limited flexion range due to adhesion of the grafted lesion **(B)**.The patient could write with a pen using the thumb’s opposition due to the irradiated fourth FDS tendon reconstruction **(C)**. The overall functional rating was 21 according to MSTS rating system. The patient developed local recurrence on the surgical margin at 20 months after surgery **(D)**.

The tumors were located in the dorsal aspect of the hand (two patients), palm (one patient), and volar aspect of the wrist (one patient). All tumors were deep-seated with maximum gross dimension of at least 5 cm. The histological diagnoses were clear cell sarcomas (two patients), synovial sarcoma (one patient), and angiosarcoma (one patient). There were three patients with AJCC stage III disease and one with stage IV disease, who had multiple pulmonary metastases. All four patients received neoadjuvant chemotherapy. One patient underwent postoperative radiation therapy (Case 2). The surgical treatment and functional results are summarized in Table 2. The surgical margin was considered to be wide in two patients and marginal in the other two. Skin flap was used for wound closure in two patients. Tumors located on the dorsal wrist (Case 1: Figure 1 and Case 2) were resected followed by reconstruction of extensor tendons. Although collapse of the carpal bones was noted after 20 months (Case 1: Figure 3A), no serious complication occurred. In the two patients whose tumors were located on the palm and volar wrist (Case 3: Figure 4 and Case 4), flexor tendons were reconstructed. The mean MSTS score was 22 (range, 21 to 25). Lower scores were seen in patients with reconstruction of flexor tendons (mean MSTS, 19.5: Cases 3 and 4) than extensor tendons (mean MSTS, 24.5: Cases 1 and 2). Two patients with AJCC stage III disease developed distant metastases at mean follow-up of 13 months (Cases 1 and 4). In Case 1, during resection of the axillary lymphadenopathy to determine possible metastasis at 19 months after surgery, we also performed biopsy of the irradiated extensor tendon to examine the histological features with patient’s informed consent (Figure 5B). There was no remarkable adhesion around the irradiated tendon, and fibroblast-like cells were seen within the strands of collagen that appear to be viable on histology (Figure 5C,D). No local recurrence was detected in the irradiated tendon, but the patient died of pulmonary metastasis at 28 months after lymphadenectomy.

**Table 2:** **Clinical results of the patients**

**Case**	**Surgical margin**	**Skin flap**	**Reconstruction**	**MSTS**	**Local recurrence (months)**	**Metastasis (months)**	**Outcome**
1	Wide	+	EDC, carpal bone	24	−	Axillary lymph node (19)	DOD
2	Marginal	−	APL, EPB, EPL, ECR-L	25	−	-	CDF
3	Marginal	+	FDP	21	+ (20)	Lung (0)	DOD
4	Wide	−	FDP	18	−	Kidney (7)	DOD

MSTS, musculoskeletal Tumor Society; EDC, extensor digitorum communis; APL, abductor pollicis longus; EPB, extensor pollicis brevis; EPL, extensor pollicis longus; ECR-L, extensor carpi radialis longus; FDP, flexor digitorum profundus; DOD, died of disease; CDF, continuous disease-free.

The type of surgical management and status of surgical margin have significant impacts on local control of soft tissue sarcoma. In the wrist and hand, the tendons, nerves, and blood vessels are adjacent to each other. Due to a lack of anatomic compartmentalization and tendency of the tumor to spread rapidly, limb salvage operations have difficulty in obtaining negative surgical margins [6]. Hence, amputation is necessary to acquire a curative surgical margin [7]. However, the functional outcome tends to be poor [7,13]. Additionally, double or single ray amputation has poorer esthetic outcomes than limb salvage operation. As a result, both the surgeon and patient are often faced with the dilemma of preserving limb function at the expense of negative surgical margins or of obtaining negative surgical margins by carrying out an amputation [6].

For functional upper limb salvage, reconstruction of vital structures should be performed following tumor resection. Complex functions of the hand are directly affected by the sacrifice of important structures when the tumor is resected [4]. The primary reconstruction of soft tissues may account for good functional results [14]. Some patients with hand soft tissue sarcoma need reconstruction of their musculoskeletal structures after resection of these tissues to obtain better functional results.

Several surgical procedures have been reported for the reconstruction of tendon defects after tumor surgery [1,4,8,9,15]. Tendon reconstruction can be accomplished by using tendons harvested from free fasciocutaneous flaps, such as the radial forearm, lateral arm, long toe extensors, gracilis muscle, or latissimus dorsi muscle [1,4].

We have applied reconstruction of musculoskeletal defects with intraoperative extracorporeal autogenous irradiated bone and tendon grafts for bony and tendon defects after resection of malignant tumors [8,9]. The advantage of this method is the potential to achieve a good hand function. The patients’ own ligaments can be restored to the original shape, size, and site. Hughes TMD *et al*. [16] reported two soft tissue sarcoma patients with metastases due to iatrogenic implantation within the donor site of the graft. Implantation of the tumor cells at the time of surgery is thought to be one of the causes of local recurrence [16]. Our method does not require additional dissection for tendon harvest; hence, contamination of the tumor cells can be avoided.

In the previous studies, no local recurrences from the irradiated grafts were reported [8,10]. Tumor sterilization was achieved clinically with a bolus of 50 Gy radiation, which is equivalent to the effect of approximately 100 Gy in partition [17]. One of four patients developed local recurrence outside the irradiated graft because the intended marginal margin to preserve the function was insufficient considering the concurrent pulmonary metastasis.

We reconstructed the extensor tendons in two of four patients and the flexor tendons in the remaining two patients after tumor resection. The MSTS scores were lower in patients with reconstruction of the flexor tendons than those with extensor tendon reconstruction. Adhesion formation of the grafted flexor tendon was one of the main reasons for insufficient recovery of hand function.

We performed histological examination to determine viability of the irradiated tendon by the use of biopsy sample. While the mechanical strength of this tendon was unknown, the histological appearance was indicative of regeneration and revascularization of the irradiated tendon.

Limb salvage operation using the irradiated tendons has better esthetic outcomes than amputation, can provide an effective alternative to amputation, and can lead to acceptable outcome for selected patients with wrist and hand soft tissue sarcoma.

## Conclusions

In this report, we successfully performed limb salvage operation for soft tissue sarcoma located on the wrist and hand using the irradiated tendons and bones. This method may be a better procedure for limb salvage operation of sarcomas on the wrist and hand than amputation.

## Abbreviations

AJCC: American Joint Committee on Cancer
MSTS: Musculoskeletal Tumor Society.

## References

[1] Saint-Cyr M, Langstein HN (2006). Reconstruction of the hand and upper extremity after tumor resection. J Surg Oncol..

[2] Siegel R, DeSantis C, Virgo K, Stein K, Mariotto A, Smith T (2012). Cancer treatment and survivorship statistics, 2012. CA Cancer J Clin..

[3] Okunieff P, Suit HD, Proppe KH (1986). Extremity preservation by combined modality treatment of sarcomas of the hand and wrist. Int J Radiat Oncol Biol Phys..

[4] Muramatsu K, Ihara K, Yoshida K, Tominaga Y, Hashimoto T, Taguchi T (2013). Musculoskeletal sarcomas in the forearm and hand: standard treatment and microsurgical reconstruction for limb salvage. Anticancer Res..

[5] Buecker PJ, Villafuerte JE, Hornicek FJ, Gebhardt MC, Mankin HJ (2006). Improved survival for sarcomas of the wrist and hand. J Hand Surg Am..

[6] Pradhan A, Cheung YC, Grimer RJ, Peake D, Al-Muderis OA, Thomas JM (2008). Soft-tissue sarcomas of the hand: oncological outcome and prognostic factors. J Bone Joint Surg Br..

[7] Bray PW, Bell RS, Bowen CV, Davis A, O’Sullivan B (1997). Limb salvage surgery and adjuvant radiotherapy for soft tissue sarcomas of the forearm and hand. J Hand Surg Am..

[8] Araki N, Myoui A, Kuratsu S, Hashimoto N, Inoue T, Kudawara I (1999). Intraoperative extracorporeal autogenous irradiated bone grafts in tumor surgery. Clin Orthop Relat Res..

[9] Ozaki R, Hamada K, Emori M, Omori S, Joyama S, Naka N (2010). Limb salvage operation using intraoperative extracorporeal autogenous irradiated bone and tendon graft for myxoid liposarcoma on dorsum of foot. Foot..

[10] Hatano H, Ogose A, Hotta T, Endo N, Umezu H, Morita T (2005). Extracorporeal irradiated autogenous osteochondral graft: a histological study. J Bone Joint Surg Br..

[11] Greene FL, Page DL, Fleming ID, Fritz AG, Balch CM, Haller DG (2002). American Joint Committee on Cancer; Cancer staging manual.

[12] Enneking WF, Dunham W, Gebhardt MC, Malawar M, Pritchard DJ (1993). A system for the functional evaluation of reconstructive procedures after surgical treatment of tumors of the musculoskeletal system. Clin Orthop Relat Res..

[13] Puhaindran ME, Rohde RS, Chou J, Morris CD, Athanasian EA (2011). Clinical outcomes for patients with soft tissue sarcoma of the hand. Cancer..

[14] Kawai A, Hasizume H, Sugihara S, Morimoto Y, Inoue H (2002). Treatment of bone and soft tissue sarcomas of the hand and wrist. Int Orthop..

[15] Talbot SG, Mehrara BJ, Disa JJ, Wong AK, Pusic A, Cordeiro PG (2008). Soft-tissue coverage of the hand following sarcoma resection. Plast Reconstr Surg..

[16] Hughes TM, Thomas JM (2000). Sarcoma metastases due to iatrogenic implantation. Eur J Surg Oncol..

[17] Inoue T, Hirabayashi Y, Mitsui H (1995). Survival of spleen colony-forming units (CFU-S) of irradiated bone marrow cells in mice: evidence for the existence of a radioresistant subfraction. Exp Hematol..

